# Septochoanal polyp with osseous metaplasia: a case report

**DOI:** 10.1186/s13256-016-0952-1

**Published:** 2016-06-03

**Authors:** Chakapan Promsopa

**Affiliations:** Department of Otolaryngology Head and Neck Surgery, Prince of Songkla University, Songkhla, 90110 Thailand

**Keywords:** Septochoanal, Choanal, Polyp, Osseous metaplasia, Calcification

## Abstract

**Background:**

Polyps originating from the posterior septum with choanal extension, also known as “septochoanal polyps,” are uncommon, and septochoanal polyps with central calcification are extremely rare. We report the second case of septochoanal polyps with central calcification in the English literature.

**Case presentation:**

A 55-year-old Thai woman presented with a progressive left-side nasal obstruction. An examination of her nose revealed an irregular lobulated mass, yellow in color, with a smooth surface that arose from her posterior nasal septum and extended down to her nasopharynx. Computed tomography revealed a large choanal mass with a central ossified structure. A punch biopsy was performed and microscopic examination showed an inflammatory polyp. The mass was removed using an endoscopic surgery technique, and the histology of this lesion confirmed a typical presentation of choanal polyps.

**Conclusions:**

Although septochoanal polyps with osseous metaplasia are known to be very rare, physicians should be aware of them and include them in the differential diagnosis of choanal mass with central calcification lesions.

## Background

Choanal polyps can be defined as benign, solitary, soft tissue masses, which extend towards the nasopharynx, or even down to the oropharynx. Most choanal polyps arise in the lateral walls of the nasal cavity, particularly the antrochoanal polyp, which originates from the maxillary sinus antrum. Some unusual origins, such as the sphenoid sinus, ethmoid sinus, middle turbinate, inferior turbinate, and nasal septum, have also been reported in the literature [1]. Choanal polyps have both cystic and solid forms, and a variety of features such as glassy and edematous; however, the central ossified type of this polyp is extremely unusual. Although the pathogenesis is still unknown, the most widely discussed pathogenetic theories involve multiple factors within the inflammation process [2]. The most common presenting symptom is nasal obstruction, either unilateral or bilateral. Other complaints are anosmia, rhinorrhea, sinusitis, snoring, and dysphagia [3]. Complete surgical removal is the standard treatment [4]. Choanal polyps originating from the posterior septum are relatively rare, and the term “septochoanal polyp” was first mentioned in a report by Birkent *et al.* [5]. There is only one reported case in the English literature in which a histological examination revealed a choanal polyp with a central ossified structure [6]. Here we describe the second such case; we discuss the clinical presentation, radiological findings, management, and the histology, and review the literature.

## Case presentation

**Fig. 1:**
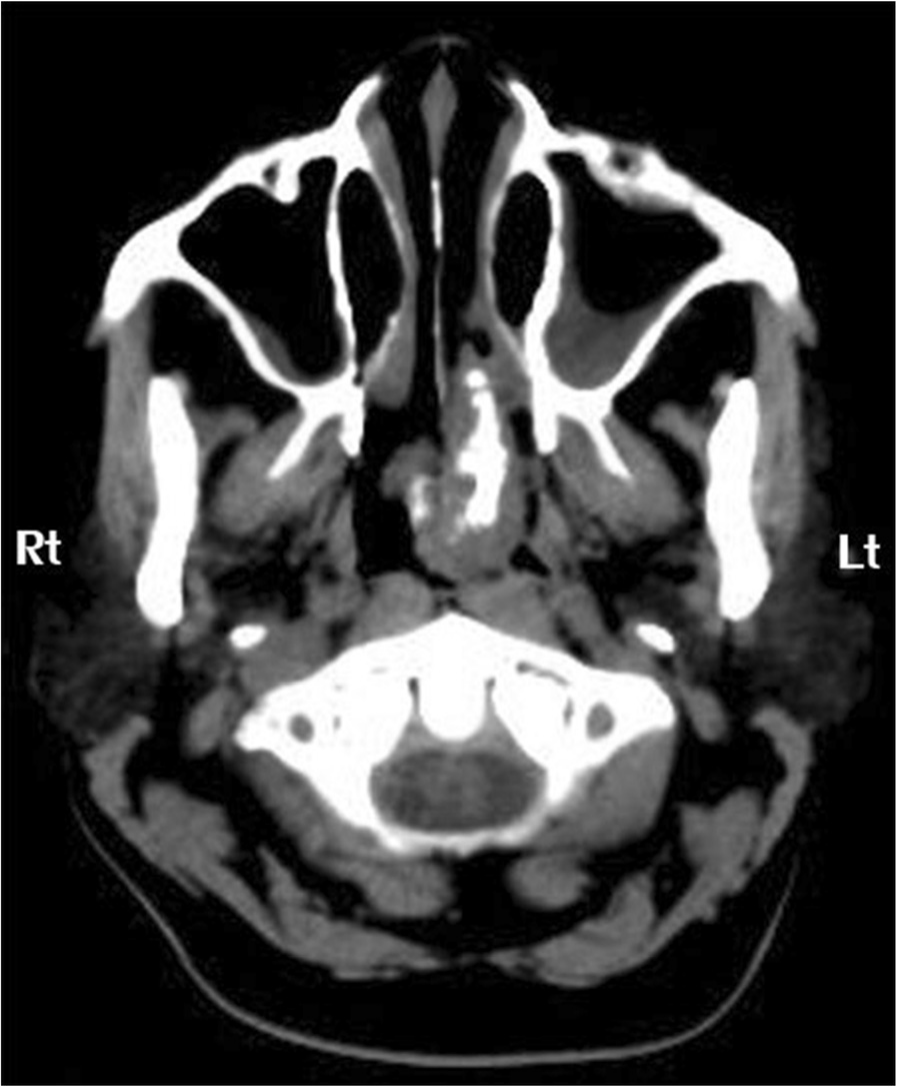
Axial view computed tomography soft tissue window demonstrated a 5×3×3 cm lobulated non-enhancing mass in posterior nasal cavity and nasopharynx arising from the left posterior nasal septum. Dense central calcification is noted. *Lt* left, *Rt* right

**Fig. 2:**
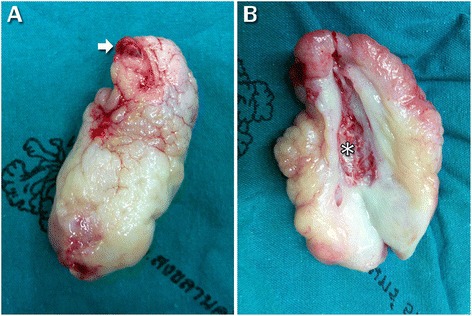
Surgical specimen. **a** The choanal polyp measured 5×3×3 cm. The irregular lobulated mass was white-yellow in color. The pedicle is shown at the upper end (*arrow*). **b** The mass contains central osseous metaplasia (*)

**Fig. 3:**
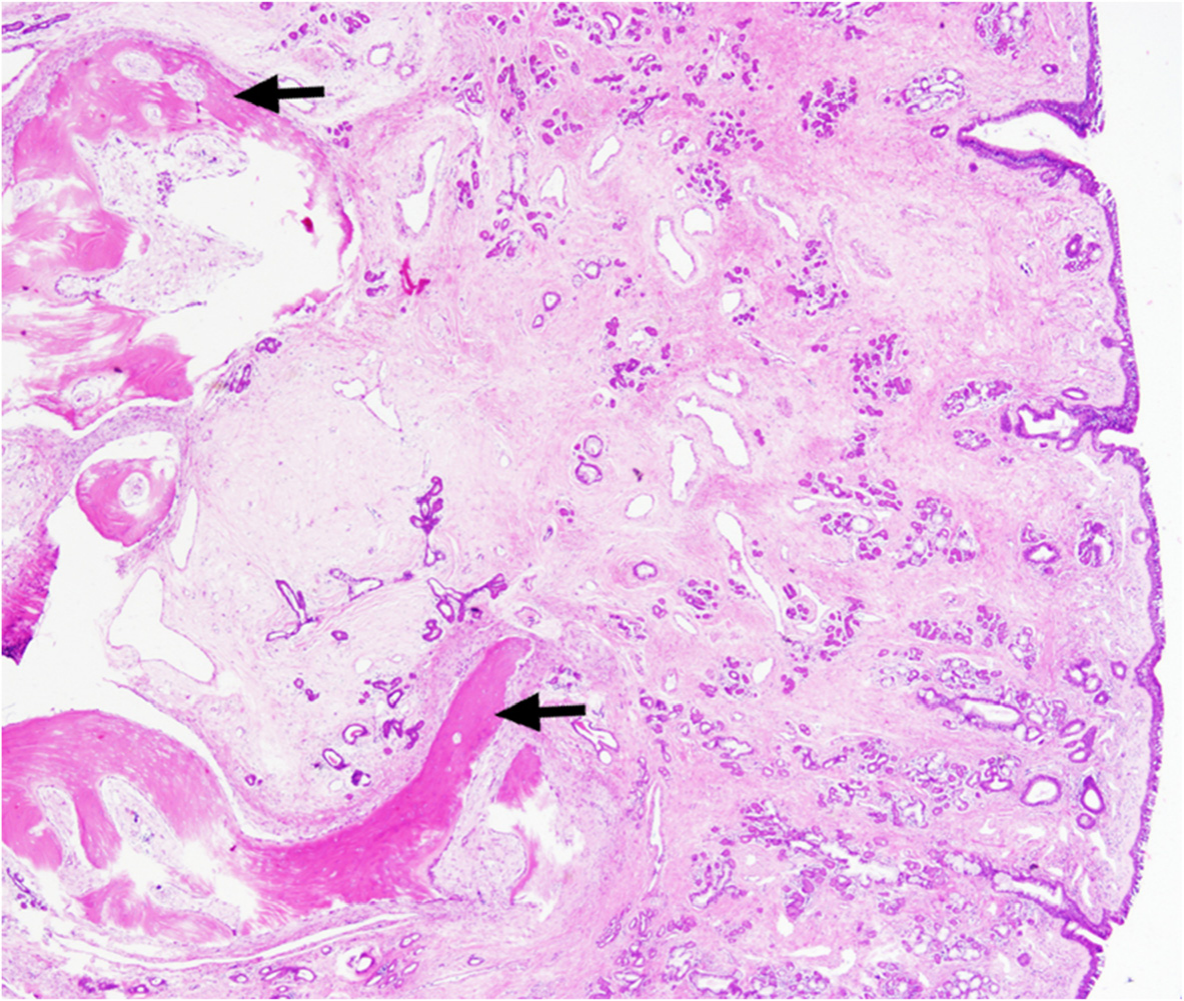
Histopathology (hematoxylin and eosin; original magnification, ×40) revealed mature trabecular bone tissue (*arrows*) covered with respiratory polypoid mucosa without eosinophils

A 55-year-old Thai woman had a 3-month history of progressive, left-side nasal obstruction. She had no history of nasal discharge, anosmia or any sign of allergy. She was otherwise healthy. A physical and nasal endoscopic examination revealed an irregular lobulated mass, yellow in color, with a smooth surface that arose with a thin pedicle from the left-side of her posterior nasal septum. This mass extended down to her nasopharynx and crossed over to the right-side of the choana. An axial view computed tomography soft tissue window demonstrated a mass with a central ossified structure at the posterior septum, which filled her nasopharynx. There was mucosal thickening in her left maxillary sinuses, while the aeration of her other paranasal sinuses was normal (Fig. 1). A punch biopsy, under local anesthesia, was performed and a microscopic examination showed an inflammatory benign polyp. During an operation, the pedicle of the polyp was identified, and excised by using endoscopic instrumentation on the left-side of her posterior septum. There was minimal bleeding from the base of the lesion and this was stopped by using monopolar cauterization. As the diameter of the mass was larger than the diameter of the choana, it was removed via the oropharynx. Pathological inspection of the surgical specimen revealed a piece of gray, brown, polypoid firm tissue that was 5×3×3 cm (Fig. 2). A histological examination revealed a mature trabecular bone tissue covered with respiratory polypoid mucosa without eosinophils (Fig. 3). At a 3-month follow-up she was symptom-free and there was no evidence of recurrence.

## Discussion

The first description of choanal polyps was reported by Killian in 1906; Killian defined them as an isolated, solitary paranasal sinus mass or cyst, which protrudes into the boundaries between the nasal cavity and the nasopharynx, or even down to the oropharynx [7]. According to Lopatin *et al*., these polyps can arise from the maxillary sinus (antrochoanal polyps), sphenoid sinus (sphenochoanal polyps), and ethmoid sinus (ethmochoanal polyps) [1]. There were other reports of unusual choanal polyps from turbinate, uncinate process, and nasal septum [8]. The most common are antrochoanal polyps, which occur as 4 to 6 % of adult polyps and 33 % of childhood polyps [9]. While sphenochoanal and ethmochoanal polyps are uncommon, septochoanal polyps are rare. The first report of choanal polyps arising from the nasal septum was published by Bailey in 1979 [10]. The case was a 38-year-old man who was found to have a nasopharyngeal mass during an examination using mirrors. The polyp in this case was approximately 15-mm long, and was a broad-based polypoid lesion without a clear stalk. Histological examination of the lesion showed multiple foci of chronic inflammatory cells. The second case was reported by Ozgirgin *et al*. in 2003 [2]. In this case the polyp was found within a 45-year-old man; he had a choanal polyp with the pedicle base arising from his posterior nasal septum that measured 6×4×2 cm. Histological examination showed that the stroma beneath the epithelium was loose and mucoid. It contained benign mucus glands, some of which exhibited cystic dilatation, as well as a small amount of mononuclear inflammatory cell infiltration.

Hakan Birkent *et al*. first mentioned the term “septochoanal polyps” with the third reported case in 2009 [5]. In this case a 52-year-old woman had a polyp originating from the superior aspect of her posterior nasal septum on the left side, with a 5.5-cm long thin pedicle. It was hanging through the choana with its bulky portion situated in her nasopharynx; its total length was 11 cm. Histopathologic examination showed the polyp with edematous stroma covered by respiratory epithelium. Hyun and Kyung reported the fourth case in 2014 [11]. Again the patient was a 52-year-old woman; she had septochoanal polyps with the pedicle base arising from her posterior nasal septum, but this case contains no details of the histopathologic result.

The first report of a septochoanal polyp with central calcification was published by Woo Sung Kim in 2010 [6]. This case involved a 43-year-old female, who had 4x3x5 cm septochoanal polyps with the pedicle base arising from the posterior nasal septum. Histopathologic showed the respiratory epithelium infiltrated with subepithelial mononuclear inflammatory cells. Osseous metaplasia (OM) is the replacement of heterotopic normal bone tissue in soft tissue. How this ectopic ossification occurs is unknown. However, one highly probable theory is that the mesenchymatous pluripotent cells of mucosal polyps are differentiated into osteoblast progenitors under the influence of bone morphogenetic proteins (BMPs) and transforming growth factor-β1 (TGF-β1); then, osteogenic signal stimulations lead to the maturation of osteoblast progenitors into osteoblasts, which can induce bone matrix secretion [12]. Most cases of OM were reported in benign colonic and endocervical polyps, and are an extremely uncommon finding in nasal polyps [13]. On computed tomography, nasal polyps are usually seen as homogeneous soft tissue masses with smooth convex margins; whereas, OM appears, on computed tomography, as multiple clustered densities seen in the center of the polyp. When faced with findings of bone within a mass of nasal polyposis, the differential diagnosis must include rhinolith, mycetoma, inverted papilloma, chondrosarcoma, osteosarcoma, and fibrosis lesions, because they can mimic nasal polyps with OM on computed tomography [14].

For this present case, we performed a punch biopsy, under local anesthesia, for diagnosis; a pathological examination showed an inflammatory benign polyp. After endoscopic excision, a histological examination confirmed atypical polyp: mature trabecular bone tissue covered with respiratory polypoid mucosa without eosinophils. From the prognosis standpoint, the key to successful treatment of septochoanal polyps is the same as for other choanal polyps [9]: the complete removal of the polyp, particularly the origin, to prevent recurrence. A septochoanal polyp has a good prognosis because the pedicle origin is easy to localize and remove completely.

## Conclusions

We reported the case of a 55-year-old Thai woman who presented with an uncommon nasal mass. Physicians should be aware, at the time of treating, of a nasal mass with central calcification arising from the posterior septum. A septochoanal polyp with OM could be the differential diagnosis. An endoscopic approach with complete surgical removal of the polyp and its pedicle origin is the standard treatment.
